# LP-OPTIMA: A Framework for Prescriptive Maintenance and Optimization of IoT Resources for Low-Power Embedded Systems

**DOI:** 10.3390/s24072125

**Published:** 2024-03-26

**Authors:** Alexios Papaioannou, Asimina Dimara, Charalampos S. Kouzinopoulos, Stelios Krinidis, Christos-Nikolaos Anagnostopoulos, Dimosthenis Ioannidis, Dimitrios Tzovaras

**Affiliations:** 1 Centre for Research and Technology Hellas, Information Technologies Institute, 57001 Thessaloniki, Greece; adimara@iti.gr (A.D.); kouzinopoulos@iti.gr (C.S.K.); krinidis@mst.ihu.gr (S.K.); dimitrios.tzovaras@iti.gr (D.T.); 2 Management Science and Technology Department, Democritus University of Thrace (DUTH), 65404 Kavala, Greece; 3 Intelligent Systems Laboratory, Department of Cultural Technology and Communication, University of the Aegean, 81100 Mytilene, Greece; canag@aegean.gr

**Keywords:** prescriptive maintenance, low-power embedded systems, IoT, resources’ optimization, lightweight machine learning, anomaly detection, malfunction detection

## Abstract

Low-power embedded systems have been widely used in a variety of applications, allowing devices to efficiently collect and exchange data while minimizing energy consumption. However, the lack of extensive maintenance procedures designed specifically for low-power systems, coupled with constraints on anticipating faults and monitoring capacities, presents notable difficulties and intricacies in identifying failures and customized reaction mechanisms. The proposed approach seeks to address the gaps in current resource management frameworks and maintenance protocols for low-power embedded systems. Furthermore, this paper offers a trilateral framework that provides periodic prescriptions to stakeholders, a periodic control mechanism for automated actions and messages to prevent breakdowns, and a backup AI malfunction detection module to prevent the system from accessing any stress points. To evaluate the AI malfunction detection module approach, three novel autonomous embedded systems based on different ARM Cortex cores have been specifically designed and developed. Real-life results obtained from the testing of the proposed AI malfunction detection module in the developed embedded systems demonstrated outstanding performance, with metrics consistently exceeding 98%. This affirms the efficacy and reliability of the developed approach in enhancing the fault tolerance and maintenance capabilities of low-power embedded systems.

## 1. Introduction

The widespread adoption of low-power embedded systems has resulted in an important shift in the rapidly evolving field of modern technology, radically changing the way electronic devices operate and interact with their environment [1]. Embedded systems are currently the backbone of a wide range of applications, from consumer electronics to industrial automation and even further, since they can easily be integrated into daily life. The idea of low-power embedded systems, a technical advancement driven by the quest for efficiency, sustainability, and extended operational lifespan, is at the core of this breakthrough trend [2]. In contrast to their conventional counterparts, which often place higher priority on processing capacity alone, low-power embedded systems carefully balance energy efficiency with usability.

The fundamental advantage of low-power embedded systems is their ability to meet the increasing demand for energy-efficient solutions in a variety of fields [3]. In the field of consumer electronics, where extended battery life and portability are critical aspects, these technologies facilitate the creation of wearables, smartphones, and other smart devices that are easily integrated into our daily lives. Furthermore, low-power embedded systems are essential for process optimization and energy conservation in industrial environments where dependability and efficiency are essential [4]. These systems serve as the basis for networks that are connected in the context of the Internet of Things (IoT), allowing devices to exchange data efficiently while consuming less energy. This offers novel opportunities in the fields of smart city development, healthcare, and environmental monitoring, in addition to improving the sustainability of IoT applications [5].

There are several significant limitations and challenges with the current methods of maintenance and resource optimization for low-power embedded systems that require awareness [6]. Initially, it should be noted that there are currently no comprehensive maintenance procedures designed especially for low-power systems [6]. The accuracy needed to anticipate and prevent faults in these energy-efficient systems is frequently lacking in current approaches, which could result in increased maintenance costs and downtime. Another significant concern is monitoring capabilities, as current instruments are unsuitable for identifying anomalies or performance degradation in advance [7]. This restriction could cause a delay in responding to potential problems, which would be harmful to their overall reliability.

Furthermore, it remains challenging to handle common problems and malfunctions in low-power systems, as response mechanisms may not be tailored to the specific power-efficient features of these systems [8]. Another challenge is the complex nature of low-power system failure detection, where current techniques often fail to produce fast and reliable identification [9]. The challenge of seamlessly integrating user activities into the resource management framework is difficult, and as low-power embedded systems expand in a variety of applications, scaling problems emerge. Furthermore, the environment is made more complex by the absence of defined protocols and procedures, which requires coordinated efforts to create a unified framework for resource optimization and prescriptive maintenance [10]. Overcoming these obstacles is critical to improving the scalability, efficiency, and dependability of low-power embedded systems.

The maintenance of such devices is essential in the rapidly evolving world of today’s technology. A new era of prolonged battery life, portability, and process optimization has been brought about by the widespread use of low-power embedded systems across a range of industries. However, regardless of their importance, there are certain noticeable shortcomings in the way these systems are currently maintained and resource-optimized, especially when considering the IoT as a whole. The lack of extensive maintenance procedures designed specifically for low-power systems, in conjunction with constraints in anticipating faults and monitoring capacities, presents notable difficulties and intricacies in identifying failures and customized reaction mechanisms, as previously discussed. The aforementioned challenges indicate the need for an integrated attempt to establish a consistent framework for resource optimization and prescriptive maintenance.

In response to the identified challenges in maintaining and optimizing low-power embedded systems, this paper proposes an innovative approach to prescriptive maintenance and resource optimization. Within the broader context of the IoT, the suggested approach seeks to address the gaps in current resource management frameworks and maintenance protocols for low-power systems. Exploiting available data streams, the approach employs a bi-fold procedure to identify potential malfunctions. It then adopts a proactive approach, ensuring that issues are resolved promptly by either informing the end-user or by setting automatic corrective steps to “cure” the failure. Furthermore, the present study stresses the importance of increasing the lifespan of these systems and proposes periodic prescriptions intended to extend the operational life of low-power embedded systems as part of the prescriptive maintenance plan. By incorporating these elements into the approach, this paper not only addresses the immediate challenges but also contributes to the overall resilience and sustainability of low-power systems. The main novelties of this work are:

- A bifold procedure, leveraging available data streams to identify potential malfunctions in low-power embedded systems;
- A proactive stance in handling potential malfunctions by implementing corrective measures;
- The proposal of a prescriptive maintenance strategy that includes periodic prescriptions to address and mitigate issues before they escalate;
- A novel resource optimization framework;
- The integration of user activities into the resource management framework enhances the overall usability and adaptability of low-power embedded systems.

Moreover, for the evaluation of the proposed prescriptive maintenance and resource optimization approach, three different novel, autonomous reduced-instruction-set-computer-(RISC)-based embedded systems have been specifically designed and developed.

The remainder of the paper is structured as follows: Section 2 presents a thorough analysis of studies, articles, and pertinent work that has been previously conducted in this field. Section 3 presents the architecture of the proposed embedded systems and the methodology of the paper, which involves the implementation of a bifold procedure for identifying malfunctions in low-power embedded systems, along with proactive measures and periodic prescriptions to address issues. Finally, the experimental results section (Section 4) involves a setup of the suggested methodology utilizing various low-power system configurations along with the evaluation results of the proposed methodology.

## 2. Related Work

A prescriptive edge-to-edge IoT network management architecture with micro-services was proposed in [11] for multiple smart edge devices. The proposed architecture utilized: the collection and processing of sensors’ raw data by edge artificial-intelligence-(AI)-based lightweight services; the monitoring of the edge and IoT network performance while considering various automated self-healing actions; and the detection of potential and fatal errors on edge devices as well as IoT entities, including sensors, actuators, and devices, using long short-term memory (LSTM) and autoencoder (AE) machine learning (ML) networks. A convolutional neural network (CNN), CNN-based LSTM autoencoder ML method was presented in [12] to detect anomaly points in different operating parameters of a Raspberry Pi, to ensure that the edge device will be operating smoothly and that the end-user will be informed to take some actions to prevent breakdowns. Five different parameters were studied: central processing unit (CPU) temperature and usage, disk and virtual memory usage, as well as power consumption.

A predictive maintenance system for Industry 4.0 using ML methods on the edge was presented in [13]. The system, executed on a Raspberry Pi, acquired simulated voltage, current, and light intensity data from a programmable logic controller (PLC) controller through message queuing telemetry transport (MQTT). The random forest method was subsequently used on the collected data to predict error values. An embedded platform for monitoring and predictive maintenance of mining motors was presented in [14]. The platform was based on the STM32F429 MCU, a MAX2085 IC for RS-485, and a DP83848C PHY chip for Ethernet communication. To perform motor fault diagnosis at the edge, based on the characteristic frequency and the current signal of the motor, a combination of a Butterworth filter, a fast Fourier transform (FFT) method, and envelope spectrum analysis was used. A similar platform for the predictive maintenance of coal mine decelerators based on vibration acceleration, vibration speed, and temperature was detailed in [15].

Moreover, significant work exists in the literature on low-power embedded systems. The initial design of a miniaturized, autonomous embedded system for environmental sensing was presented in [16]. The system was based on a Cortex-M0 microcontroller unit (MCU) with the AEM10941 PMIC, the BME680 environmental sensor, and the RSL10 and SX1261 ICs for bluetooth low energy (BLE) and LoRa wireless communication, respectively. An autonomous LoRaWAN node for environmental monitoring of buildings was presented in [17]. The node used a LoRa SX1276 transceiver, together with an Ambiq Apollo2 MCU based on a Cortex-M4 core, a BME680 environmental sensor, and a Fujitsu ferroelectric random access memory (FRAM). The node was programmed to make periodic environmental measurements, including temperature, relative humidity, and air pressure, at 10-minute intervals using BME680; the results were transmitted wirelessly via SX1276 with a spreading factor of SF7BW125 and +8 dBm RF output power, allowing the coverage of a small building. An autonomous BLE node was presented in [18]. The node used the ST BLUENRG-2 BLE SoC as a load, together with an STM32L0-based MCU from ST for ambient light sensing. A system with very similar characteristics and the addition of ST HTS221 for moisture and temperature sensing was detailed in [19]. An autonomous BLE sensor node designed for the monitoring of physical parameters, including temperature, humidity, and resistivity, in reinforced concrete was presented in [20]. The autonomous node was designed to be embedded into a concrete cavity and could be powered using wireless power transmission. The transmission of the measurements was performed via the QN9080 BLE SoC.

Table 1 summarizes the related work on error detection methods and maintenance strategies followed. The terminology used includes distinctions related to the embedded system or edge device, the integration of sensors, the power characteristics, the employed maintenance strategy, the monitoring level, and the detection method. The “embedded system” refers to the underlying hardware architecture, with options such as a Raspberry Pi (Rpi) or a custom designed system, like the ones proposed in this work. The term “integrated sensor” refers to sensors embedded in the system. A “sensor node” refers to discreet sensors connected wirelessly to the system.

As it may be observed from this section, the present work is distinct in all respects. It utilizes the capabilities of a customized embedded system, operates in a low-power setting, applies prescriptive maintenance, and monitors integrated sensors as well as the edge. This work varies from the presented publications in that it addresses a wide range of topics, including system architecture, power efficiency, maintenance strategy, and monitoring techniques. With an emphasis on low-power systems and effective sensor integration, this level of detail positions the work being presented as a comprehensive and cutting-edge solution for edge-to-edge IoT network management.

## 3. Methodology

The methodology used for the resource management system involves a comprehensive approach to optimizing system performance on low-power autonomous embedded systems. First, the architecture and dependencies of three different such systems, designed and developed specifically for this work, are presented. Then, the proposed resource management system is extensively detailed.

### 3.1. Architecture and Dependencies of Autonomous, Low-Power Embedded Systems

**Figure 1:**
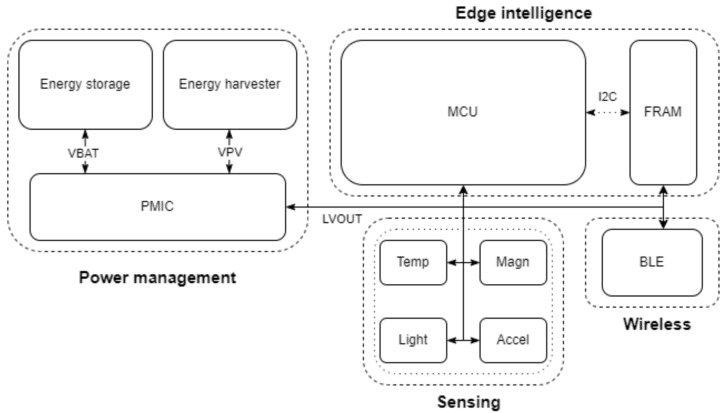
High-level architecture of the embedded systems.

For the work of this paper, three novel, autonomous embedded systems have been designed and developed, with an emphasis on low power consumption. Each system consists of an ARM MCU, the MB85RC64TAPN-G-AMEWE1 non-volatile FRAM for data storage, the BME680 environmental sensor for temperature and humidity measurements, and the RSL10 BLE System-on-Chip (SoC) for short-range wireless communication. For energy generation, distribution, and storage, the following components were used: An AEM10941 PMIC from E-peas, two EXL1-1V20-SM cells by Lightricity with a total active surface of 2 cm^2^ and the Powerstream GEB201212 battery with a capacity of 10 mAh. All sensing and communication integrated circuits (ICs) are connected to the MCU via the I2C interface. An overview of the common architecture layer of the proposed embedded systems is depicted in Figure 1.

The systems utilized different MCUs in 100-pin packages, each based on a different processor core, as listed in Table 2.

All three MCUs feature brown-out reset (BOR) circuitry, a feature designed to handle temporary drops in the power supply voltage. When the voltage drops below a certain threshold but does not completely reach zero, it can lead to erratic behavior or data corruption in the system. The BOR circuit monitors the power supply voltage, and when it falls below a specified level, it triggers a reset of the microcontroller or device.

After a system or a power reset, the MCUs begin execution in run mode, or CRun mode in the case of STM32H743VG. In these modes, full power is provided via the embedded voltage regulators (or by an external power supply where applicable) to the core domain ($V_{CORE}$), commonly consisting of the CPU, the digital peripherals, and the memories. Moreover, multiple low-power execution modes exist to reduce power consumption during idle periods, with different compromises each in terms of consumption, startup time, and number of available wake-up sources, as detailed below.

Different errors in software and hardware can cause different exceptions to occur, including the following [21,22]:

- **HardFault**. Generic fault conditions existing for all classes of fault that cannot be handled by any of the other exception mechanisms. Typically, it is used for unrecoverable system failures
- **MemManage**. handles memory protection faults that are determined by the memory protection unit (MPU) or by fixed memory protection constraints for both instruction and data memory transactions.
- **BusFault** handles memory-related faults, other than those handled by the MemManage fault, for both instruction and data memory transactions. Typically, these faults arise from errors detected on the system buses.
- **UsageFault**. handles non-memory-related faults caused by instruction execution. A number of different situations cause usage faults, including undefined instruction, invalid state on instruction execution, error on exception return, word or halfword memory accesses to an unaligned address, or division by zero.

Specific technical characteristics of each MCU are detailed below.

#### 3.1.1. STM32L496VG MCU

The STM32L496VG MCU is a Cortex-M4 core based on the ARMv7E-M architecture with a maximum frequency of 80 MHz, a 1 MB flash and 320 KB of SRAM. It has a 25 nA quiescent current and 37 μA/MHz energy consumption in run mode when a switched mode power supply (SMPS) is used.

Apart from the run mode, the MCU implements the following low-power modes [23]: sleep mode with CPU clock off and all peripherals being able to run and wake up the MCU; low-power run mode when the system clock frequency is reduced below 2 MHz; low-power sleep mode entered from the low-power run mode; three different stop modes that achieve the lowest power consumption while retaining the content of SRAM and registers; standby mode that allows to achieve the lowest power consumption with BOR; and shutdown mode allows to achieve the lowest power consumption without BOR.

The microcontroller features three types of reset, where “reset” refers to the process of returning to a predefined initial state of the embedded system:

- Power reset
- System reset
- Backup domain reset

A power reset is generated as a consequence of BOR as well as when exiting from standby or shutdown modes. BOR sets all registers, apart from the registers of the backup domain, to their respective reset values. When exiting standby mode, all registers in the $V_{CORE}$ domain are set to their reset value. Registers outside the $V_{CORE}$ domain are not impacted. When exiting shutdown mode, a BOR is generated, resetting all registers except those in the backup domain.

A system reset sets most registers to their reset values. It is generated when, among others: an external reset occurs (via the NRST pin); a window or independent watchdog event occurs; or a software reset occurs. Each of these works by pulling the NRST line low for 20 μs. A software reset can be triggered via the NVIC_SystemReset() system function that sets the SYSRESETREQ bit of the application interrupt and reset control register (AIRCR) (see [24] for more details). The control/status register (RCC_CSR) is set by the hardware to indicate the cause of the software reset.

The MCU features a backup domain that includes an LSE oscillator, an RTC, 32×32-bit backup registers of the RTC, and a RCC backup domain control register (RCC_BDCR). The backup registers (RTC_BKPxR) are not reset by system reset or when the device wakes up from standby mode. A backup domain reset can be used to set all registers of the backup domain to their reset values. This type of reset can be generated through software triggered by setting the RCC_BDCR register.

#### 3.1.2. STM32H743VG MCU

The STM32H743VG MCU is based on the much more powerful Cortex-M7 core and the ARMv7E-M architecture. It has a CPU frequency of up to 480 MHz, a 2 MB flash, and 1 MB of RAM; it includes a double-precision FPU and 32 Kb of L1 cache. It has a hefty power consumption in comparison to the Cortex-M4 cores, requiring approximately 275 μA/MHz during run mode.

The system operating mode depends on the CPU subsystem modes (CRun, CSleep, CStop), the D2 domain modes (DRun, DStop, DStandby), and the system (D3) autonomous wakeup domain [25]. The CPU subsystem modes are: CRun, where the CPU and CPU subsystem peripherals are clocked; CSleep, where the CPU clocks are stalled and the CPU subsystem peripherals are clocked; and CStop, where the CPU and CPU subsystem peripheral clocks are stalled. For the D1/D2 domain modes: DRun, where the domain bus matrix is clocked; DStop, where the domain bus matrix clock is stalled; and DStandby, where the respective domain is powered down. For the D3 domain, the system can be in either run, stop, or standby mode.

The STM32H743VG MCU features the following types of reset:

- Power-on/off reset
- System reset
- Local resets

The power-on/off reset is activated when the input voltage ($V_{DD}$) is below a threshold level. This is the most complete reset since it resets the whole circuit, except the backup domain. The system reset is similar to that of the M4 core above. For the local resets, the CPU can reset itself via the CPURST bit in the RCC_AHB3RSTR register. Moreover, some resets also depend on the domain status, as detailed in [25]. The RCC_RSR register can be used to determine the cause of the reset.

#### 3.1.3. STM32U5A5VJT MCU

The STM32U5A5VJT MCU is based on the Cortex-M33 core and the ARMv8-M architecture, including a single-precision FPU. It packs significantly more performance characteristics compared to STM32L496VG, with a CPU frequency of up to 160 MHz, 4 MB of flash memory, and 2.5 MB of SRAM. At the same time, it is more energy efficient than the M4 cores, with approximately 18.5 μA/MHz of energy consumption in low-power run mode. The STM32U5A5VJT MCU features identical modes and reset types to STM32L496VG [26].

### 3.2. Resource Management

**Figure 2:**
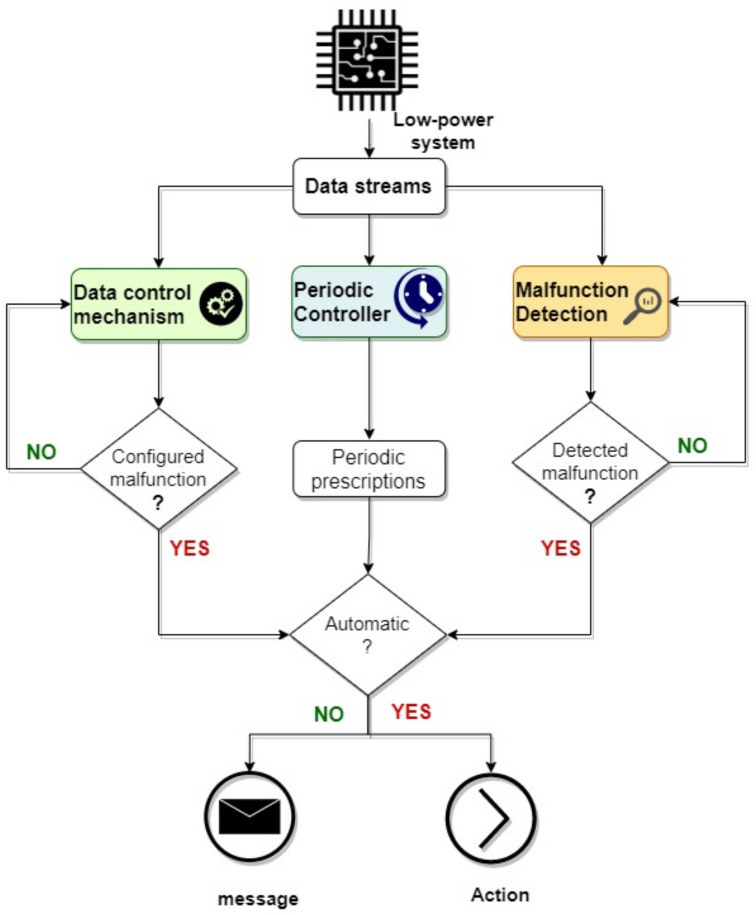
Resource management flow chart.

The resource management layer in the low-power system plays a crucial role in optimizing system performance, minimizing latency, reducing energy consumption, and proactively detecting potential malfunctions. This layer is designed to efficiently monitor and manage system resources through a combination of services, applications, and tools (Figure 2).

The data control mechanism (DCM) is an essential process that performs real-time assessment of available data streams and is at the center of this layer. This process, which is guided by particular system settings and dependencies, determines decisions based on data analysis insights. Its primary objective is to analyze data streams and detect the existence of issues. When an issue is detected, the system takes immediate action, either by warning the user or by performing automated healing actions. The malfunction detection method (MD), which uses AI methods for sophisticated anomaly identification, is a complementary component of the data control system. The process makes use of specific information to conduct a thorough analysis of data streams. The objective is to identify anomalies that might indicate a system vulnerability. When a malfunction is detected, the user is alerted or executes automated healing actions.

Finally, the periodic controller (PCTRL) process operates in cycles to maintain the continuous health and functionality of the system. This part does two functions: it notifies users of potential periodic activities that need to be performed and, when needed, it operates independently to execute preventive actions. The system may sustain stability and efficiency over time by combining periodicity with proactive actions, offering a proactive approach to system management and user interaction.

### 3.3. Data Control Mechanism

DCM incorporates several key features to ensure efficient and optimized system performance. Firstly, it is programmed to prevent power consumption from surpassing the lowest threshold by actively monitoring resource usage. Furthermore, the system is equipped with awareness of connections to both autonomous and non-autonomous components. Its capability is targeted at checking the presence or absence of connections with these components. The mechanism leverages the presence or absence of these links to inform its decision-making process. In Table 3, the overall description of the DCM is illustrated. The description is used to track the specific malfunctions or issues monitored by the DCM, each marked as dcmNumber indicating the respective issues. The malfunction/ issue describes the problem, while the type indicates the type of action. Each prescription (i.e., DCM prescription deciphered dcm) might be an automated action performed directly from the DCM, called “ACT” or a prescription that is sent to the user as a message, called “MSG”.

### 3.4. Periodic Controller

The periodic controller (PCTRL), integrated as another fundamental component within the resource management system, operates as an essential element of the prescriptive maintenance strategy. PCTRL is specifically designed to implement periodic prescriptions while offering efficiency in resource management. This process plays a crucial role in ensuring the continuous health and efficiency of the low-power system. Periodic prescriptions encompass a range of proactive actions and maintenance tasks that contribute to the prevention of potential issues and the optimization of overall system performance. The PCTRL automatically generates prescriptions that are considered suitable for automated implementation as part of its automated capabilities. By implementing routine activities, these automated prescriptions maintain the stability of the system. Moreover, the PCTRL generates prescription (PC) notifications for prescriptions that need end-user intervention or actions that cannot be completed automatically. End users then receive these messages, which include brief and precise instructions on what has to be done.

Table 4 outlines the periodic prescriptions generated by the PCTRL. It includes an action (ACT) for a monthly software reset to maintain system stability (`pc1’) and a message (MSG) for biannual updates and upgrades, notifying users about the scheduled process (`pc2’). Moreover, the system automatically sends a prescription to the user, serving as a timely reminder for the necessity of updating the system (`pc3’). To securely update the system firmware, wolfBoot [27] can be used, an open-source secure bootloader for Cortex-M cores that can be integrated into the system by partitioning the on-board flash memory. Monthly wolfBoot updates are seamlessly integrated into the system, ensuring optimal performance and security with each iteration (`pc4’). Finally, the pc5 prescription serves as a reminder to users that a periodic system update has been successfully performed. This proactive communication ensures transparency and reassures users about the ongoing maintenance efforts to keep the system running smoothly. Table 4 provides clarity on the types of prescriptions, their frequencies, and the specific actions or messages associated with each.

Periodical resets for the low-power system are essential to ensuring the reliability and stability of the microcontroller’s operations. Periodic resets allow the system to revert to a known state, therefore decreasing the likelihood of problems like data corruption or accumulated errors. This procedure contributes to sustained performance by preserving the integrity of the microcontroller’s operations and averting extended operations that could result in problems. Periodic upgrades are also essential for introducing new features, security advancements, and improvements, keeping the low-power system up to date and optimized for changing problems and requirements. The low-power system’s long-term dependability and functioning are supported by frequent upgrades and resets.

### 3.5. Malfunction Detection

The MD module is an element operating upstream of DCM. It plays a pivotal role in the early detection of operational errors, identifying the implemented system as a proactive system. This module acts as a preventive mechanism, enhancing the system’s ability to identify and address anomalies before they affect data control processes. Its proactive nature significantly contributes to the overall robustness and reliability of the system by preemptively mitigating potential malfunctions.

This module employs a lightweight AE model designed to promptly detect potential malfunctions within the system. In Table 5, an overview of the examined malfunctions is provided, along with corresponding prescriptions (md) communicated to the user. The assessment involves four operational parameters associated with heap and stack memory, RAM memory, current power consumption, and CPU cycle count.

#### 3.5.1. Autoencoder

AE is a type of neural network designed to acquire a condensed representation from input data, functioning as an unsupervised learning method that leverages self-supervised training techniques. Comprising an input layer, an output layer, an encoder, and a decoder neural network, along with a latent space, this architecture involves the encoder compressing input data from the input layer into the latent space and, subsequently, the decoder decompressing it for transmission to the output layer.

The primary goal of the autoencoder is to reduce the dimensions of input data while maintaining essential information regarding the data structure. Specifically, given an input $x\in R^{m}$, the encoder compresses *x* into an encoded representation z = $e(x)\in R^{n}$. The decoder then reconstructs this representation into an output $x^{\prime }=d\left(z\right)\in R^{m}$. The autoencoder is trained by minimizing the reconstruction error, defined by the equation:

(1) $$L=\frac{1}{2}\sum_{x}{∥x-x^{\prime }∥}^{2}.$$

The LSTM autoencoder, convolutional autoencoder, and vanilla autoencoder are only a few of the various autoencoder types that have been suggested in the literature. The LSTM autoencoder consists of LSTM modules in both the encoder and decoder modules. LSTMs are well suited for time series forecasting or anomaly detection due to their ability to learn patterns in data over long sequences [28]. In general, an encoder-decoder model in anomaly detection applications learns the representation of the data using only the normal sequences, as stated in [29], and then reconstructs the data using the trained model. When the model is fed with an abnormal sequence, it might not be reconstructed well, leading to a high error.

#### 3.5.2. AE-LSTM

**Figure 3:**
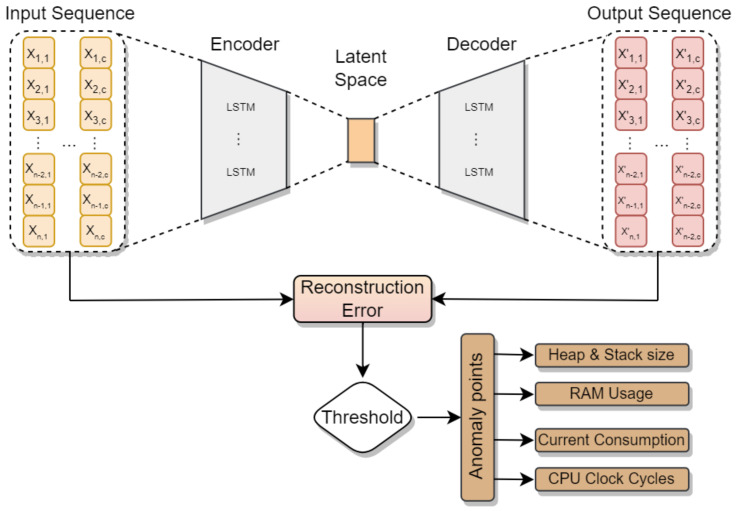
Anomaly detection method based on Reconstruction Errors (AE-LSTM).

Figure 3 describes the proposed AE-LSTM. Sequences of $x\in R^{n*c}$ are used as input in the proposed method, where *n* is the number of instances in time *t* and *c* is the number of features for each instance. The input consists of metrics recorded every second, including heap and stack size, the number of CPU clock cycles, RAM usage, and current power consumption in mA.

Before being fed into the AE, the input data (i.e., heap and stack size, the number of CPU clock cycles, RAM usage, and current power consumption) undergoes a preprocessing phase. This involves replacing missing values with previous values, and a scaling method is applied to bring features that are not in percentage form (CPU clock cycles and current power consumption) into a specific interval. Missing values in a dataset are instances where no data value is stored for the variable in an observation. Specifically, the approach to dealing with missing values involves replacing them with the most recent values [30] when they are less than 25% of the data within the predefined monitoring granularity. This method addresses potential data loss due to various errors, such as connection or power issues, ensuring the integrity and continuity of the dataset for the AE analysis. This decision is made on the basis that large gaps in the data, more than 25% missing values, would significantly degrade the quality and accuracy of the data analysis [31]. If the gap is greater than this threshold, it is omitted. By clearly defining this threshold and the associated action of omitting data exceeding this gap, it is ensured that the autoencoder and any subsequent analyses are based on reliable and robust data. This strategy improves the model’s ability to identify anomalies while ensuring the data fed into the model is of high quality and consistency, enhancing the overall reliability of the system’s monitoring and predictive maintenance capabilities.

The standardization method was used to shift the distribution of data to have a mean of zero and a standard deviation of one. The following equation describes the method:

(2) $$x_{scaled}=\frac{x-mean}{StandardDeviation}$$

Subsequently, the scaled data is then transformed into [samples, time steps, and features] in order to be used as input in the AE.

The encoder layer can be described in detail using the following equation:

(3) $$h_{i}=f_{\theta }\left(x_{scaled}\right)=s\left(\sum_{n}^{j=1}W_{ij}^{input}x_{scaled}j+b_{input}\right),$$

where $x_{scaled}$ is the input vector with $x_{scaled}\in R^{n*c}$, θ is the parameter $\left\{W^{input},b^{input}\right\}$, *W* is the encoder weight matrix with dimension m∗d, (m<d), and *b* is the bias.

Following this, the decoder layer can be described as follows:

(4) $$x_{i}^{\prime }=g_{\theta }^{\prime }\left(h\right)=s\left(\sum_{n}^{j=1}W_{ij}^{hidden}h_{j}+b_{hidden}\right),$$

where the parameter set of the decoder is $\theta =\{W^{hidden},b^{hidden}\}$.

LSTM, introduced by Hochreiter and Schmidhuber in 1997 [32], represents a distinctive form of recurrent neural network (RNN). Comprising interconnected units at each level, it features one or more memory cells, along with input, output, and forget gates. The fundamental concept of LSTM is outlined as follows:

(5) $$i^{t}=\sigma \left(W^{i}x_{scaled}^{t}+V^{i}h^{t-1}+b^{i}\right),$$

(6) $$f^{t}=\sigma \left(W^{f}x_{scaled}^{t}+V^{f}h^{t-1}+b^{f}\right),$$

(7) $$o^{t}=\sigma \left(W^{o}x_{scaled}^{t}+V^{o}h^{t-1}+b^{o}\right),$$

(8) $$c^{t}=f^{t}⊙c^{t-1}+i^{t}⊙\tanh\left(W^{c}x_{scaled}^{t}+V^{c}h^{t-1}+b^{c}\right),$$

(9) $$h^{t}=o^{t}⊙\tanh\left(c^{t}\right),$$

where *t* is the time step, $h^{t}$ the hidden state at time *t*, $x_{scaled}^{t}$ the data at time *t*, $h^{t-1}$ the hidden state at previous time, $i^{t}$ the input gate, $f^{t}$ the forget gate, $o^{t}$ the output gate, and $c^{t}$ is a memory cell. Additionally, $W\in R^{d*k}$, $V\in R^{d*d}$, $b\in R^{d}$, σ is the sigmoid function, ⊙ denotes the element-wise product, and *k* is a hyper-parameter that represents the dimensionality of hidden vectors.

In the context of the AE-LSTM architecture described in Figure 3, a seamless integration of AE principles and LSTM is achieved. The encoder layer encapsulates the essence of the AE by compressing the input data into a latent representation. This encoded information is then further processed by the LSTM layer, facilitating the capture of temporal dependencies and intricate patterns within the sequential data. Subsequently, the decoder layer reconstructs the information from the hybridized representation. The LSTM, with its input, forget, and output gates, effectively contributes to both learning and generating sequences based on the compressed features derived from the AE. This approach, leveraging the strengths of both autoencoders and LSTMs, enriches the model’s ability to capture and reproduce intricate patterns in sequential data.

The reconstruction error serves as a metric for evaluating the fidelity of the model’s output compared to the original input sequences. The mean absolute error (MAE) was used as the metric to calculate the reconstruction error. Mathematically, the MAE for a given instance at time *t* is expressed as the average absolute difference between the input sequence $x^{\left(t\right)}$ and its reconstructed output ${\hat{x}}^{\left(t\right)}$:

(10) $${\mathrm{MAE}}^{\left(t\right)}=\frac{1}{c}\sum_{i=1}^{c}\left|{x_{scaled}}_{i}^{\left(t\right)}-{\hat{x}}_{i}^{\left(t\right)}\right|,$$

where *c* is the number of features in each instance.

Additionally, to classify data points as either normal or anomalous, a threshold value needed to be established. Given that the output of the MAE adheres to a normal distribution, the following equation was employed to calculate the threshold value [12]:

(11) Threshold_value=μ(MAE)+k∗σ(MAE),

where μ represents the mean of the MAE, *k* the confidence value, and σ the standard deviation of the MAE. Using the features of the normal distribution, this method defines a threshold that includes a certain proportion of the data. By fine-tuning the value of the *k*, the model’s sensitivity to anomalies can be adjusted, offering a flexible and data-driven method for establishing an effective threshold for anomaly detection.

#### 3.5.3. Implementation of AE in Low-Power Embedded Systems

Deploying machine learning models on resource-constrained microcontrollers is a challenging process, and converting a TensorFlow LSTM model to TensorFlow Lite (TFLite) is a pivotal step in this process. The proposed AE-LSTM was trained on TensorFlow version 2.14.0. The conversion to TFLite was achieved using the keras file, the TFLite Converter in Python, and unfolding each LSTM layer using unidirectional LSTM [33].

Furthermore, to enhance the performance of the exported TFLite model in terms of execution time and memory size, a quantization technique was implemented. Two distinct methods for neural network quantization were considered: post-training quantization (PTQ) and quantization-aware training (QAT) [34]. The primary distinction between PTQ and QAT lies in the stage at which the scale is computed. In PTQ, the quantized model is derived after the network has been trained and is typically constrained to FP16 or INT8 quantization. On the other hand, in QAT, the quantized model is computed during the training phase, preserving significantly more accuracy in the results compared to PTQ. For this study, QAT and 8-bit integers were specifically chosen, with the 8-bit integer being the lowest supported value by the library. Finally, the fine-tuned TFLite model was integrated into the microcontroller using the X-CUBE AI 8.1.0 module provided by STM [35].

## 4. Experimental Results

In the results section, a comprehensive analysis of the experimental findings, encompassing both real and simulated datasets within the embedded systems context, is presented. The results focus on MD, showcasing the efficacy of the proposed methodology. The outcomes demonstrate robust performance in accurately identifying and addressing malfunctions, validating the practical applicability of our approach to enhancing the reliability of embedded systems.

### 4.1. Experiment Setup and Results

**Figure 4:**
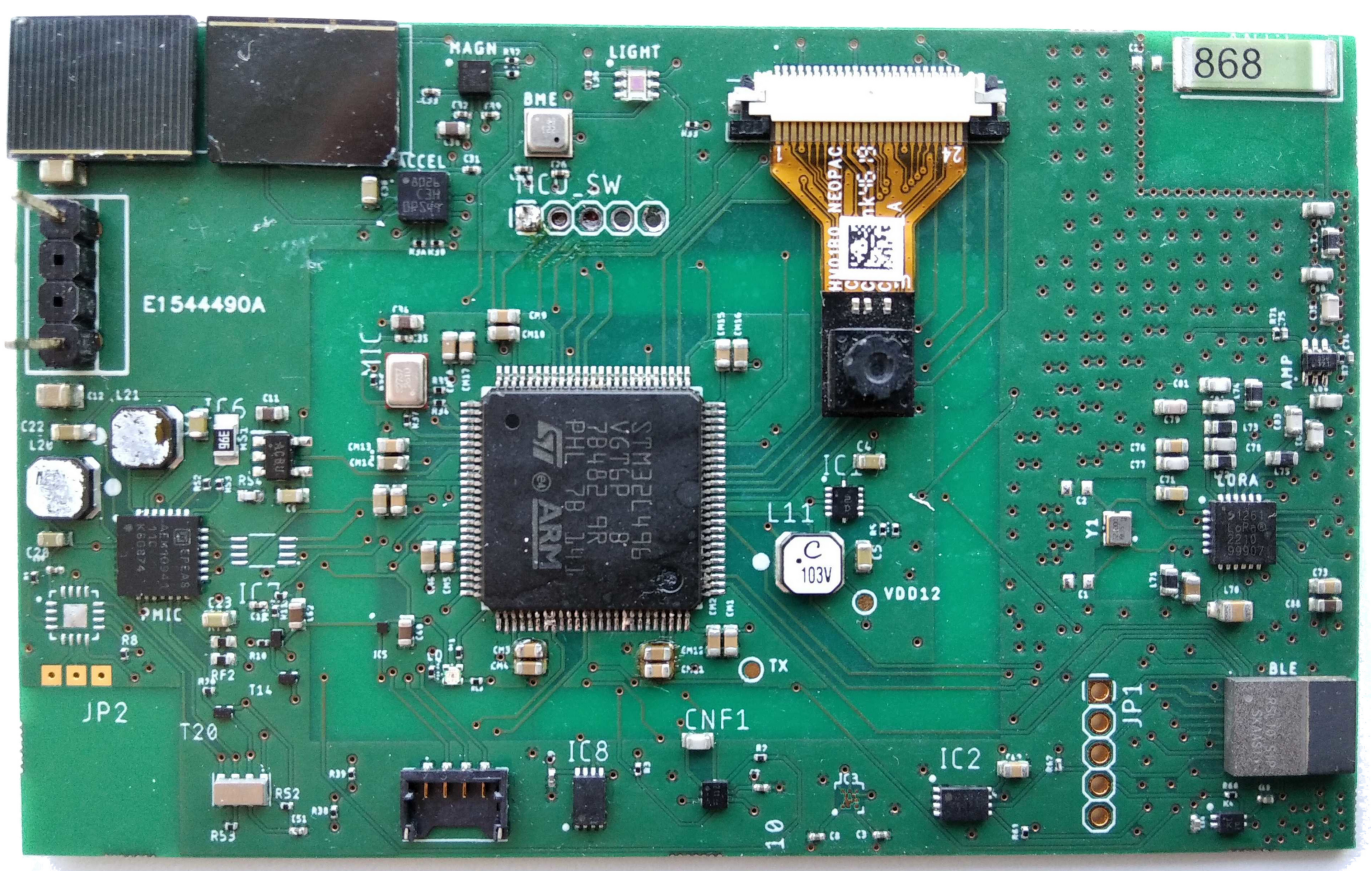
Prototype of the miniaturized embedded system based on the Cortex-M4 core.

The evaluation of the proposed methodology was conducted on three different autonomous, miniaturized embedded systems. The schematic of the evaluation system is presented in Figure 1. The three different evaluation systems utilized the STM32L496VG, STM32H743VG, and STM32U5A5VJT microprocessors, respectively. Figure 4 depicts a prototype of the embedded system utilizing the STM32L496VG MCU. The operating frequency of the MCUs was 4 MHz.

### 4.2. Data Set

Real and simulated data were created for the training and evaluation phases of the malfunction detection module. A total of 30,000 instances were collected, with 10,000 instances from each of the three different platforms. These data represented the normal operational conditions of the system and were utilized for the training process of the malfunction detection module. For the test set, 500 anomaly instances for each platform were generated using simulations based on the four scenarios described in Section 4.2.2. All instances had a granularity of one second.

#### 4.2.1. Real Data

Real data were collected from three STM32 MCUs, capturing features associated with heap and stack memory, RAM availability, current power consumption, and CPU cycle count. Heap and stack memory, along with RAM availability data, were obtained through the linker script. To measure CPU cycles, the CYCCNT register was initialized for cycle counting, and the cycle count was recorded by marking the start and end of the targeted code segment. The elapsed cycles were then calculated for a precise assessment of the computational load. Additionally, the current power consumption was collected using the INA219 sensor [36]. The heap and stack memory, as well as RAM availability, are measured in percentage (%), while the current power consumption is in milliamperes (mA), and the CPU cycle count is an integer value.

#### 4.2.2. Simulated

Four different scenarios (SC) were simulated for each monitored system feature (heap and stack memory, RAM usage, power consumption, and CPU cycle count). In each scenario, one of the four monitored features was modified to assess the effectiveness of the proposed malfunction detection algorithm. Table 6, illustrates the description of each scenario, the potential causes of malfunctions, and the system features affected by these malfunctions.

- Heap and Stack Size Error: In this scenario, the application encounters a heap and stack size error when the allocated memory during runtime exceeds the available heap space. Additionally, errors occur when the stack overflows due to extensive nesting of function calls or because of sensor input, such as receiving false values from a sensor.
- RAM Usage: In this scenario, errors occur in monitoring RAM usage when free allocated memory is not executed properly or is missing. Additionally, buffer overflows in pointers lead to the same problem with RAM usage.
- Current Consumption: In this scenario, an error occurs in current consumption when a damaged temperature sensor (BME 680) is connected to the platform.
- CPU Cycle Count: In this scenario, stack overflow is induced by recursive function calls, leading to a deviation between the normal CPU cycle count and the simulated situation. This discrepancy indicates the occurrence of an error in the system.

### 4.3. Results

In this section, the evaluation of the Malfunction Detection module is presented using real and simulated data. Additionally, there is a thorough analysis and comparison with state-of-the-art (SoA) methods, including AE with dense layers, one-class support vector machine (OC-SVM), and Isolation Forest (IF). Finally, a detailed analysis of the segmentation of each component of the proposed AE-LSTM model is presented, offering comprehensive statistics for RAM and ROM memory usage, along with the corresponding execution times and energy consumption.

#### 4.3.1. Malfunction Detection Results

The experimental results of two different variations of the AE-LSTM method (with one layer in the encoder and the decoder (1-1) and with two layers in the encoder and the decoder (2-2)) and a comparison with SoA methods are presented in Table 7. The experiments were conducted under various confidence values in each case.

In the evaluation of AE models (Dense and LSTM), three different confidence values (k) were employed, leading to different threshold values as presented in Equation (11). In the case of OC-SVM, different values of the nu parameter were employed, where nu serves as an upper bound on the fraction of margin errors and a lower bound on the fraction of support vectors relative to the total number of training examples. Distinct values for the contamination parameter were set to control the threshold for the decision function, determining when a scored data point should be considered an outlier. QAT quantization (8 bits) was used in all models. For the evaluation of the methods, precision, recall, accuracy, F1 score, and the multiply-accumulate operation (MACC) were employed as metrics to assess the performance and computational complexity of each method.

Across all confidence values, the AE-LSTM(2-2) consistently outperforms all other AE models, as well as OC-SVM and IF. Specifically, the best-performing AE-LSTM(2-2) was observed at k = 8, exhibiting precision close to 0.985, recall and accuracy close to 0.999, an F1 score close to 0.990, and a MACC of 16484. The second-best model was the AE-LSTM(1-1), showing a slight reduction in all evaluation metrics and a higher reduction in MACC, close to 78.42%. The best OC-SVM was observed at nu = 0.8, with all evaluation metrics lower by approximately 3% in each metric. However, the MACC of the OC-SVM was much higher than that of the highest AE model (AE-LSTM(2-2)), approximately 174% higher. For achieving an optimal trade-off between model size and evaluation metrics, the AE-LSTM (1-1) model was selected as the optimal.

**Figure 5:**
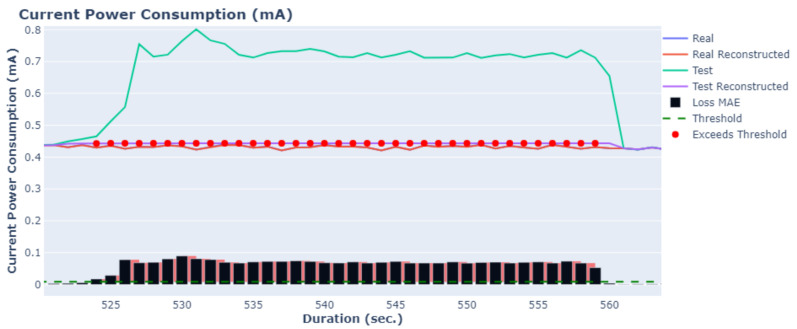
Real and reconstructed time series of the current power consumption using the AE-LSTM(1-1) model.

**Figure 6:**
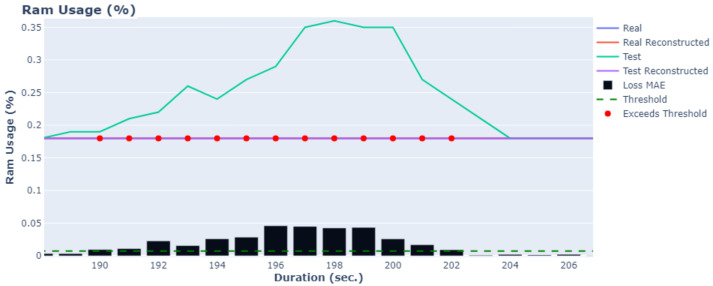
Real and reconstructed time series of the Ram usage using the AE-LSTM(1-1) model.

The results for two variables, current power consumption and RAM usage, are depicted in Figure 5 and Figure 6. The blue lines represent the actual values obtained from the microprocessors during normal operating conditions, while the orange lines show the results of the AE-LSTM(1-1) using the actual values as input. Any deviation between these two lines indicates that the retrieved values are considered anomalies, signifying potential malfunctions.

The green line represents simulated data used as the test set, which includes anomaly points. The purple lines illustrate the reconstructed lines using the AE-LSTM(1-1). Additionally, bar plots for each variable present the reconstruction error using the MAE metric, along with lines indicating the threshold value (dashed green). This threshold is selected using the three-sigma statistical rule.

As observed, the proposed method is capable of identifying the majority of anomaly points at the onset of the error phase. Specifically, on Figure 5 at 524 s, an increase in the loss MAE is observed, indicating that errors begin to occur. At this point, the loss MAE exceeds the predefined threshold, and these points are annotated as anomaly points. Around 560 s, the error starts to diminish, indicating that it is resolved. Consequently, the loss MAE decreases, and the values fall within the predefined threshold. Similar results are observed in Figure 6; from 190 s to 202 s, the loss MAE exceeds the predefined threshold and is annotated as an anomaly point. The clear distinction between actual and reconstructed values, coupled with the effective identification of anomalies, underscores the robustness and accuracy of the proposed AE-LSTM(1-1) method in anomaly detection.

#### 4.3.2. Evaluation in the Embedded Systems

The evaluation of the embedded systems involved the examination of SoA and AE anomaly detection methods across three distinct platforms: STM32L496VG (L4), STM32H743VG (H7), and STM32U5A5VJT (U5). Key metrics, including model inference processing time (Proc. Time), flash and RAM memory occupancy (%), current power consumption, and energy consumption, were utilized to assess the performance of each method on these diverse hardware platforms. Table 8 illustrates the performance analysis for each microprocessor.

In the case of the H7 microprocessor, the AE-Dense(1-1) method demonstrated the fastest inference time and the lowest energy consumption. However, a notable trade-off was observed in terms of F1-score and accuracy, which were considerably lower compared to the selected AE-LSTM(1-1) model. Despite having an execution time of approximately 13.2 milliseconds, higher than the model with the fastest inference time, the AE-LSTM(1-1) remained the preferred choice for real-time applications. This preference is attributed to the overall superior performance of the AE-LSTM(1-1), emphasizing the significance of metrics beyond inference time and energy consumption. The F1-score and accuracy metrics play crucial roles in assessing the model’s ability to accurately identify anomalies, making the AE-LSTM(1-1) more suitable for real-time solutions despite a slightly longer execution time.

Similar results were observed on the remaining two microprocessors, where the selected model exhibited an inference time slightly higher than that on the H7 platform. This minor increase in execution time can be attributed to the less favorable specifications of the other microprocessors. In terms of accuracy, F1-score, and overall anomaly detection capabilities, the AE-LSTM(1-1) remained the best solution for real-time detection.

Table 9 provides a detailed energy breakdown of the AE-LSTM(1-1) model across the three different platforms: H7, U5, and L4. The table includes the overall processing time, energy consumption, and a detailed breakdown of time and energy at different layers of the model. For the H7 platform, the model’s execution time was 13.2 ms, with the majority of energy consumed in the 1st LSTM layer of both the encoder and decoder. Similarly, for the U5 and L4 platforms, the processing time was 17.032 ms and 17.829 ms, with corresponding energy consumption of 0.039 mJ and 0.038 mJ, respectively. The breakdown reveals that the encoder and decoder 1st LSTM layers are the dominant contributors to energy consumption, emphasizing the significance of optimizing these layers for energy efficiency. The inclusion of intermediate nodes also contributes to a small fraction of energy usage.

### 4.4. System Requirements and Constraints

The comprehensive evaluation of the LP-OPTIMA framework across varying hardware specifications underscores the STM32L496VG (L4) as an essential baseline for analysis. This microcontroller, with its Cortex-M4 core, 1MB of flash memory, and 320 KB of RAM, represents the lower threshold of system capabilities within the examination scope. As the system with the smallest flash and RAM capacities among the evaluated MCUs, STM32L496VG encapsulates the minimum requirements for effective deployment of the LP-OPTIMA framework. Its operational parameters may provide a testbed for assessing the framework’s performance under constrained resources. Moreover, the framework’s energy consumption metrics on the STM32L496VG highlight its capacity to maintain energy efficiency in resource-limited environments. This is pivotal for low-power embedded systems, where power conservation is paramount. Finally, by establishing the STM32L496VG (L4) as the baseline, invaluable insights into the framework’s scalability are gained. Performance improvements observed as we transition to more capable MCUs (STM32H743VG and STM32U5A5VJT) validate the framework’s adaptability to a broader spectrum of hardware platforms. All the above are summarized in Table 10 based on results and information presented in Table 2 and Table 8.

## 5. Conclusions

This research addresses the challenges of maintenance and resource optimization for low-power embedded systems. The proposed approach introduces a trilateral framework involving periodic prescriptions, automated control mechanisms, and an AI malfunction detection module. The evaluation of the AI malfunction detection module, particularly the AE-LSTM(1-1) model, demonstrated outstanding performance across various metrics, with precision, recall, accuracy, and F1 score consistently exceeding 98%. The results of the MD module were compared with state-of-the-art methods, including AE with dense layers, OC-SVM, and Isolation Forest.

The evaluation extended to real-life testing on three different embedded systems based on various ARM Cortex cores. The selected AE-LSTM(1-1) model demonstrated superior performance in terms of accuracy, F1 score, and overall anomaly detection capabilities, making it the preferred choice for real-time applications despite a slightly longer execution time. The research also provided a detailed breakdown of energy consumption for the AE-LSTM(1-1) model across different hardware platforms. The results highlighted the importance of optimizing the first LSTM layers in both the encoder and decoder for energy efficiency.

Moving forward, future research can enhance the proposed framework for low-power embedded systems. Firstly, the integration of dynamic prescriptions based on real-time system conditions presents an opportunity for adaptive maintenance strategies, potentially optimizing resource utilization more effectively. The continuous refinement of machine learning models remains a crucial area of exploration. Investigating advanced techniques and architectures could lead to even more accurate and efficient anomaly detection models for low-power embedded systems. However, it is crucial to acknowledge certain limitations. The evaluation focused on three specific ARM Cortex cores, and future work should encompass a broader range of hardware platforms to ensure the generalizability of the proposed framework.

## Figures and Tables

**Table 1:** Summary of related work.

Work	Embedded System	Low Power	Maintenance Strategy	Monitoring Level	Detection Method
[11]	Rpi	NO	Prescriptive	Embedded system Sensor nodes	LSTM
[12]	Rpi	NO	Prescriptive	Embedded system Sensor nodes	LSTM
[13]	Rpi	NO	Predictive	Sensor nodes	Random Forest
[14]	Custom	YES	None	Embedded system	Butterworth filter, FFT and envelope Spectrum analysis
[15]	Custom	YES	None	Embedded system	Butterworth filter, FFT and envelope spectrum analysis
This work	Custom	YES	Prescriptive	Embedded system Sensor nodes Integrated sensors	AE-LSTM

**Table 2:** Specifications and electrical characteristics of STM32 MCUs.

Model	Core	Frequency	Flash	RAM	Shutdown	Standby	Stop	Run
STM32L496VG	Cortex-M4	80 MHz	1 MB	320 KB	25 nA	108 nA	2.57 μA	91 μA/MHz (LDO) 37 μA/MHz (SMPS)
STM32H743VG	Cortex-M7	480 MHz	2 MB	1 MB	-	2.95 μA	290 nA	275 μA/MHz
STM32U5A5VJT	Cortex-M33	160 MHz	4 MB	2.5 MB	150 nA	195 nA	2 μA	18.5 μA/MHz

**Table 3:** Data control mechanism monitoring and prescriptions.

Number	Malfunction/Issue	Type	Prescription
dcm1	Over current consumption threshold	ACT	System reset
dcm2	Over current consumption threshold after restart	MSG	{“alertType”: “System Alert”, “message”: “Overcurrent Threshold Exceeded After Restart. Please check”.}
dcm3	No connection to sensor node	ACT	System reset
dcm4	No connection to sensor component	ACT	System reset
dcm5	No connection to sensor node after restart	MSG	{“alertType”: “System Alert”, “message”: “No connection to sensor node A after restart. Please check”.}
dcm6	No connection to integrated sensor after restart	MSG	{“alertType”: “System Alert”, “message”: “No connection to integrated sensor A after restart. Please check”.}
dcm7	Erratic behavior or data corruption	MSG	{“alertType”: “System Alert”, “message”: “System voltage too low. Increase voltage!”}
dcm8	Drops in the power supply voltage below threshold	ACT	System reset
dcm9	Restart due to BOR	MSG	{“alertType”: “System Alert”, “message”: “System restarted. Data corruption prevented!”}
dcm10	Error during execution of System software (UsageFault)	MSG	{“alertType”: “System Alert”, “message”: “System restarted. Error prevented”.}
dcm11	Bus Memory fault (BusFault)	MSG	{“alertType”: “System Alert”, “message”: “System memory fault. Check firmware or data execution”}
dcm12	Bus Memory access violation (MemManage)	MSG	{“alertType”: “System Alert”, “message”: “SMemory access violation. Please check firmware”.}
dcm13	Bus unrecoverable system failures (HardFault)	MSG	{“alertType”: “System Alert”, “message”: “System restarted due to unrecoverable error. Please check!”}

**Table 4:** Periodic Controller prescriptions.

Description	Type	Frequency	Prescription
pc1	ACT	Monthly	Software reset to maintain system stability.
pc2	MSG	Biannual	{“alertType”: “Prescription”, “message”: “Periodic system update and upgrade scheduled. Please prepare for the process”.}
pc3	MSG	Variable	{“alertType”: “Prescription”, “message”: “Please update you system”.}
pc4	ACT	Monthly	wolfBoot
pc5	MSG	Monthly	{“alertType”: “Prescription”, “message”: “Periodic system update performed”.}

**Table 5:** Malfunction Detection Prescriptions.

Code	Malfunction/Issue	Prescription Type	Prescription
md1	Insufficient Heap and Stack Memory	MSG	{“alertType”: “System Alert”, “msg”: “Insufficient Heap and Stack Memory detected. Please check and optimize.”}
md2	Low RAM Availability	MSG	{“alertType”: “System Alert”, “msg”: “Low RAM detected. Please check and optimize”.}
md3	High Current Power	MSG	{“alertType”: “System Alert”, “msg”: “High Current Power Consumption. Please check sensors”.}
md4	Unusual CPU Cycle Count	MSG	{“alertType”: “System Alert”, “msg”: “Unusual CPU Cycle Count. Please check and optimize”.}

**Table 6:** Simulated scenarios for system errors.

	sc1	sc2	sc3	sc4
**Descr.**	Function call nesting or overflow is occurred due to input of a sensor	Uninitialized variables or buffer overflows	Unexpected power-related issues	Count deviating from expected clock cycle values
**Possible** **Causes**	• Dynamic memory aaallocation • Recursive function calls	• Failure to free aaallocated memory • Buffer overflows in aaarrays or pointers	• Incorrect peripheral aaor sensor configurations • Hardware issues	• Recursive function calls • Incorrectly configured aainterrupt priorities
**Features** **Affects**	Heap and Stack memory	Ram Usage	Power Consumption	CPU Cycle Count

**Table 7:** Comparison of the proposed AE-LSTM model with SoA.

Confidence Value	Model	Precision	Recall	Accuracy	F1 Score	MACC
k = 6	AE-Dense (1-1)	0.919	0.985	0.964	0.955	420
	AE-Dense (2-2)	0.925	0.998	0.978	0.965	1636
	AE-LSTM (1-1)	0.940	0.998	0.978	0.965	3556
	AE-LSTM (2-2)	0.945	0.998	0.997	0.974	16,484
nu = 0.005	OC-SVM	0.918	0.945	0.956	0.945	45,267
Contamination = 0.002	IF	0.915	0.935	0.946	0.935	1144
k=7	AE-Dense (1-1)	0.949	0.985	0.965	0.974	420
	AE-Dense (2-2)	0.949	0.985	0.965	0.974	1636
	AE-LSTM (1-1)	0.965	0.998	0.998	0.984	3556
	AE-LSTM (2-2)	0.975	0.998	0.998	0.990	16,484
nu = 0.05	OC-SVM	0.938	0.955	0.966	0.956	45,267
Contamination = 0.003	IF	0.935	0.955	0.955	0.945	1144
**k = 8**	AE-Dense (1-1)	0.949	0.985	0.965	0.974	420
	AE-Dense (2-2)	0.955	0.995	0.965	0.979	1636
	AE-LSTM (1-1)	0.985	0.998	0.998	0.985	3556
	**AE-LSTM (2-2)**	**0.985**	**0.999**	**0.999**	**0.990**	**16,484**
nu = 0.08	OC-SVM	0.955	0.965	0.975	0.965	45,267
Contamination = 0.005	IF	0.950	0.960	0.965	0.955	1144

Bold row indicates the best-performing model. Underlined row indicates the selected model.

**Table 8:** Performance metrics of the anomaly detection models on different platforms.

Platform	Model	Proc Time (ms)	Flash Memory Occupied (%)	Ram Memory Occupied (%)	Current Power Consumption (mA)	Energy (mJ)
L4	Dense AE (1-1)	1.328 ms	1.047% (10.73 KB)	0.359% (1.56 KB)	0.635 mA	8.4 × $10^{-4}$ mJ
	Dense AE (2-2)	4.168 ms	1.585% (16.24 KB)	1.996% (6.39 KB)	0.635 mA	0.003 mJ
	AE LSTM (1-1)	17.829 ms	3.208% (32.85 KB)	0.925% (2.93 KB)	0.655 mA	0.038 mJ
	AE LSTM (2-2)	61.866 ms	8.287% (84.86 KB)	1.428% (4.57 KB)	0.657 mA	0.131 mJ
	OC-SVM	83.832 ms	12.061% (123.51 KB)	0.387% (1.24 KB)	0.687 mA	0.231 mJ
	IF	3.547 ms	4.474% (45.83 KB)	0.400% (1.28 KB)	0.697 mA	0.008 mJ
H7	Dense AE (1-1)	1.172 ms	0.536% (10.73 KB)	0.151% (1.56 KB)	4.812 mA	0.005 mJ
	Dense AE (2-2)	3.354 ms	0.812% (16.24 KB)	0.620% (6.39 KB)	4.821 mA	0.016 mJ
	AE LSTM (1-1)	13.2 ms	1.642% (32.85 KB)	0.284% (2.93 KB)	4.853 mA	0.215 mJ
	AE LSTM (2-2)	47.2 ms	4.243% (84.86 KB)	0.443% (4.57 KB)	4.876 mA	0.668 mJ
	OC-SVM	77.458 ms	6.175% (123.51 KB)	0.120% (1.24 KB)	4.878 mA	1.211 mJ
	IF	3.174 ms	2.293% (45.82 KB)	0.124% (1.28 KB)	4.865 mA	0.015 mJ
U5	Dense AE (1-1)	1.271 ms	0.268% (10.73 KB)	0.063% (1.56 KB)	0.685 mA	7.5 × $10^{-4}$ mJ
	Dense AE (2-2)	3.816 ms	0.406% (16.24 KB)	0.260% (6.39 KB)	0.685 mA	0.008 mJ
	AE LSTM (1-1)	17.032 ms	0.821% (32.85 KB)	0.119% (2.93 KB)	0.689 mA	0.039 mJ
	AE LSTM (2-2)	58.28 ms	2.121% (84.86 KB)	0.186% (4.57 KB)	0.674 mA	0.140 mJ
	OC-SVM	74.245 ms	3.082% (123.51 KB)	0.051% (1.24 KB)	0.679 mA	0.185 mJ
	IF	3.654 ms	1.143% (45.83 KB)	0.052% (1.28 KB)	0.670 mA	0.008 mJ

Underlined row indicates the selected model.

**Table 9:** Energy Breakdown of the AE-LSTM(1-1).

Platform	Layers	Proc Time (ms)	Energy (mJ)
L4	AE-LSTM (1-1)	17.829 ms	0.038 mJ
	• Encoder 1st LSTM	8.076 ms	0.017 mJ
	• Decoder 1st LSTM	9.143 ms	0.020 mJ
	• Intermediate nodes	0.609 ms	0.001 mJ
H7	AE-LSTM (1-1)	13.2 ms	0.215 mJ
	• Encoder 1st LSTM	6.095 ms	0.096 mJ
	• Decoder 1st LSTM	6.681 ms	0.113 mJ
	• Intermediate nodes	0.461 ms	0.006 mJ
U5	AE-LSTM (1-1)	17.032 ms	0.039 mJ
	• Encoder 1st LSTM	7.715 ms	0.018 mJ
	• Decoder 1st LSTM	8.734 ms	0.020 mJ
	• Intermediate nodes	0.582 ms	0.001 mJ

**Table 10:** LP-OPTIMA Framework Performance Analysis Across STM32 Platforms.

Metric/Platform	STM32L496VG (L4-Baseline)	STM32H743VG (H7)	STM32U5A5VJT (U5)
Processor Core	Cortex-M4	Cortex-M7	Cortex-M33
Frequency	80 MHz	480 MHz	160 MHz
Flash Memory	1 MB	2 MB	4 MB
RAM	320 KB	1 MB	2.5 MB
Processing Time (ms)	17.829	13.2	17.032
Flash Memory Occupied (%)	3.208%	1.642%	0.821%
RAM Occupied (%)	0.925%	0.284%	0.119%
Current Power Consumption (mA)	0.655	4.853	0.689
Energy (mJ)	0.038	0.215	0.039
Performance Insight	Optimal balance between performance and resource usage. Suitable for real-time applications with constrained resources.	Enhanced performance with increased power usage. Suitable for applications requiring faster processing and higher reliability.	Efficient energy use with minimal resource occupancy, demonstrating scalability to more powerful systems without significant energy cost.
Constraints	- Max energy: 0.04 mJ - Min processing speed: 15 ms - Max flash occupancy: 4% - Max RAM occupancy: 1%	- Max energy: 0.22 mJ - Min processing speed: 10 ms - Max flash occupancy: 2% - Max RAM occupancy: 0.3%	- Max energy: 0.04 mJ - Min processing speed: 15 ms - Max flash occupancy: 1% - Max RAM occupancy 0.2%

## Data Availability

Data are contained within the article.
